# Study of Hyperbranched Poly(ethyleneimine) Polymers of Different Molecular Weight and Their Interaction with Epoxy Resin

**DOI:** 10.3390/ma11030410

**Published:** 2018-03-09

**Authors:** Frida Román, Pere Colomer, Yolanda Calventus, John M. Hutchinson

**Affiliations:** Departament de Màquines i Motors Tèrmics, Escola Superior d’Enginyeries Industrial, Aeroespacial i Audiovisual de Terrassa, Universitat Politècnica de Catalunya, Barcelona Tech, Carrer de Colom 11, 08222 Terrassa, Spain; colomer@mmt.upc.edu (P.C.); calventus@mmt.upc.edu (Y.C.); hutchinson@mmt.upc.edu (J.M.H.)

**Keywords:** hyperbranched polymers, poly(ethyleneimine), epoxy resin, dielectric relaxations, dynamic mechanical relaxations

## Abstract

Two different commercial hyperbranched poly(ethyleneimine)s (HBPEI), with molecular weights (MW) of 800 and 25,000 g/mol, and denoted as PEI800 and PEI25000, respectively, as well as the mixtures with a Diglycidyl Ether of Bisphenol-A (DGEBA) epoxy resin, have been studied using thermal analysis techniques (DSC, TGA), dielectric relaxation spectroscopy (DRS), and dynamic mechanical analysis (DMA). Only a single glass transition is observed in these mixtures by DSC. DRS of the HBPEIs shows three dipolar relaxations: γ, β, and α. The average activation energy for the γ-relaxation is similar for all HBPEIs and is associated with the motion of the terminal groups. The β-relaxation has the same average activation energy for both PEI800 and PEI25000; this relaxation is attributed to the mobility of the branches. The α-relaxation peak for all the HBPEIs is an asymmetric peak with a shoulder on the high temperature side. This shoulder suggests the existence of ionic charge trapped in the PEI. For the mixtures, the γ- and β-relaxations follow the behaviour of the epoxy resin alone, indicating that the epoxy resin dominates the molecular mobility. The α-relaxation by DRS is observed only as a shoulder, as a consequence of an overlap with conductivity effects, whereas by DMA, it is a clear peak.

## 1. Introduction

### 1.1. Hyperbranched Polymers

Hyperbranched polymers (HBPs) belong to a class of synthetic tree-like macromolecules called dendritic polymers. Dendritic polymers are materials with a highly branched structure. Due to the large number of end groups, with one at the end of every branch, they are highly functional. These end groups offer the possibility for further modification and special applications, and the type of end group determines, to a considerable extent, the properties. The high degree of branching renders entanglement of the polymers impossible, which results in low melt and solution viscosities. In general, the dendritic architecture can be considered as consisting of six subclasses: dendrons and dendrimers, linear-dendritic hybrids, dendrigrafts or dendronised polymers, hyperbranched polymers, multi-arm star polymers, and hypergrafts or hypergrafted polymers [1]. The most prominent feature of these types of polymers is their degree of branching (DB), which defines the ratio of branched, terminal, and linear units in the polymer structure. By definition, DB is 1.0 for dendrimers and <1.0 for hyperbranched polymers (0.5 for statistical growth). The first three subclasses exhibit perfect structures with DB equal to 1.0, while the latter three exhibit randomly branched structures [2].

HBPs have special properties, which lead to their widespread industrial applications. One of their most interesting physical properties is their considerably different viscosity characteristics in comparison with their linear analogues, which is a consequence of the molecular architecture [3,4]. The architecture of an HBP depends on the structure of the monomer from which it is prepared. The structure of HBPs can be described in terms of two characteristic parameters: the DB and the average number of branches (ANB) [5,6,7,8]. Because of their highly functionalised globular structures, HBPs exhibit different properties from those of linear polymers of the same molar mass, such as low entanglement in the solid state, high solubility in various solvents, and low viscosity [9]. 

Due to their unique properties and easy synthesis, HBPs have a wide range of potential applications. HBPs and their substitutes can be used as nanomaterials for host-guest encapsulation [10,11,12], the fabrication of organic-inorganic hybrids [13,14], biomaterials applications such as bio-carriers and biodegradable materials [1,15,16,17], rheology modifiers or blend components [18,19,20,21,22,23,24], cross-linking or adhesive agents [25,26,27], dye receptive additives for polyolefins [28,29], tougheners for thermosets [30,31,32,33,34,35,36,37,38], compatibilisers [39], dispersants [40], and for supports in organic synthesis [41,42]. HBPs have been used as the base for various coating resins, such as powder coatings [43,44], high solids coatings [45], flame retardant coatings [46,47], and barrier coatings for flexible packing [48], on account of their high solubility, low viscosity, and abundant functional groups. As a consequence of their large number of functional groups and interesting optical, electrochemical, biological, and mechanical properties, the patterning of HBP films is also receiving increased attention [49,50,51,52,53,54].

**Figure 1:**
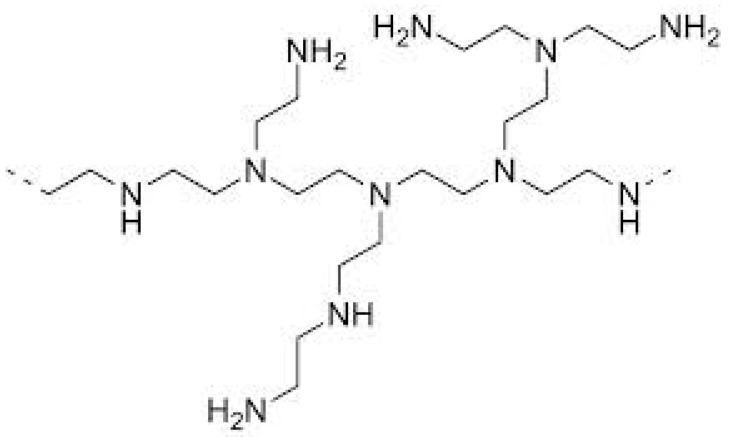
Chemical structure of HBPEI.

Recently, the use of HBPs as curing agents for thermosets has come to some prominence. HBPs can effectively crosslink epoxy resins, and this may be advantageous if these polymers exhibit improved compatibility with epoxy resins over the conventional linear aliphatic amine curing agents of low molecular weights. The reaction kinetics of a wide variety of epoxy systems with HBPs has been studied, including trifunctional [55] and tetrafunctional epoxy systems [56], anhydride cured [57] and cationically initiated epoxy systems [58], UV-cured epoxy systems for which significant improvements in toughness were reported [59,60,61], and as curing agents for nanocomposites based on a combination of epoxy resin, hyperbranched polymer, and layered silicate [62,63,64]. Recently, the use of Lupasol^®^, a hyperbranched poly(ethyleneimine) (HBPEI), as a multifunctional crosslinker of epoxy resins has been reported. HBPEI, the structure of which is shown in Figure 1, can react with epoxy monomers by an epoxy-amine condensation mechanism and is thus incorporated into the network structure of the thermoset [65], and a comparison of the curing kinetics of HBPEI with a linear aliphatic amine shows that the densely branched architecture of high molecular weight Lupasol^®^ creates significant mobility restrictions, which can have a strong effect on the reaction kinetics, and hence on the glass transition temperature and on the degree of crosslinking of the cured material [65]. The effect of the molecular weight of the PEI core when HBPEI is used as a reactive modifier in DGEBA formulations cured with 1-methylimidazole has been studied [66].

### 1.2. Dielectric Relaxation Spectroscopy of Epoxy-HBP Systems

A better understanding of the interaction between the HBP and the epoxy resin during cure, and hence of the effect of the reaction kinetics on the final network structure, may be obtained from dielectric relaxation spectroscopy (DRS), which provides information about the segmental mobility within a polymer. Such studies can be applied to both the epoxy resin and the HBP separately, as well as to their mixture and to the system during cure and in the fully cured state. There is a large body of literature devoted to the study of the evolution of the primary and secondary relaxations in various epoxy resins, in large part DGEBA, and of epoxy-based mixtures which form networks by a condensation reaction, principally with diamines.

For the DGEBA epoxy alone, a glass transition (α-relaxation) and two secondary relaxations, β and γ, have been reported [67,68,69]. The γ-relaxation in the pure epoxy and in the epoxy-amine system is related to the dynamics of dipoles localized in the epoxide end groups [68], or more generally, with the local motions of dipoles of unreacted species [70]. As regards the β-relaxation, from dynamic mechanical analysis (DMA) of anhydride cured epoxy systems, Shimbo et al. [71] assigned it to the formation of H-bonds between hydroxyl ether groups and methoxy branches [72], while Mangion and Johari [70] later, from DRS studies, assigned it to the local motion of dipolar groups that are created during the cross-linking reaction.

DRS has been used to study the molecular dynamics of a wide variety of HBPs which, in most cases, display a glass transition and two secondary relaxations, the γ-relaxation invariably being attributed to the motion of the terminal groups. In contrast, there are rather fewer studies of the molecular dynamics of cross-linking systems in which HBPs are incorporated, and of how the relaxation behaviour changes as the cross-linking reaction proceeds. Maroulas et al. [73] investigated the molecular dynamics in hyperbranched polyimides cross-linked with ethylene glycol diglycidyl ether and found that the γ-relaxation becomes slower but increases in magnitude as the cross-linking proceeds. This apparently anomalous behaviour was attributed to the sum of two opposing effects: an increase in free volume accompanied by increased constraint as a result of the crosslinking. The influence of free volume on the γ-relaxation was also suggested by the work of Zhu et al. [74], who found that the γ-relaxation temperature increased with generation number in hyperbranched polyester systems. This was interpreted in terms of a coupling of the molecular motions of the terminal groups with the α-relaxation glass transition, in other words, that the γ-relaxation was influenced by its environment.

In a previous paper [69], we studied the molecular dynamics of the following systems by DRS and DMA: the pure HBPEI with a molecular weight of 2000 (PEI2000, trade name Lupasol PR8515, BASF); the neat epoxy resin (diglycidyl ether of bisphenol-A, DGEBA); the unreacted mixture of HBPEI and epoxy, denoted ELP; the ELP system during non-isothermal cure; and the fully cured system. The objective of the present work is to determine the effect of the molecular weight of the hyperbranched polymer (HBPEI) on its glass transition and secondary relaxations, and to investigate its relaxation behaviour when incorporated into an epoxy resin matrix. For this study, we have used HBPEIs of different molecular weights, PEI800 (Lupasol^®^ FG) and PEI25000 (Lupasol^®^ WF), for comparison with our previous study with PEI2000 [69].

## 2. Results and Discussion

### 2.1. Preliminary Experiments

**Figure 2:**
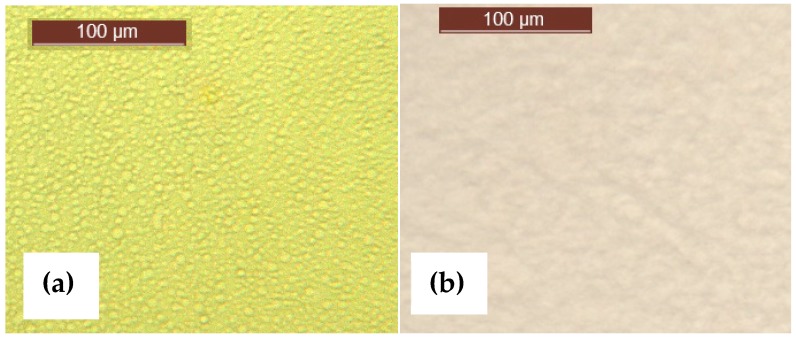
Morphology of uncured (**a**) ELP800 and (**b**) ELP25000 mixtures.

Preliminary studies by transmission optical microscopy reveal a two-phase morphology of the uncured ELP800 mixture at room temperature after degassing, as shown in Figure 2. In this mixture, droplets with a diameter between 3 and 14 μm can be distinguished. This morphology is similar to that observed earlier for the epoxy-Lupasol mixtures with an HBPEI of molecular weight (MW) 2000 g/mol [69], ELP2000, though the dimensions of the droplets here are generally larger than those for ELP2000, which were 5 μm or smaller. On the other hand, the highest molecular weight mixture, ELP25000, presents a completely different morphology, as can be seen in Figure 2. Despite this two-phase morphology in the uncured mixtures, when the system is cured, there is no further evidence of the existence of separate phases. This is indicated by Scanning Electron Microscopy images of the fracture surfaces of the fully cured samples (not shown here).

**Figure 3:**
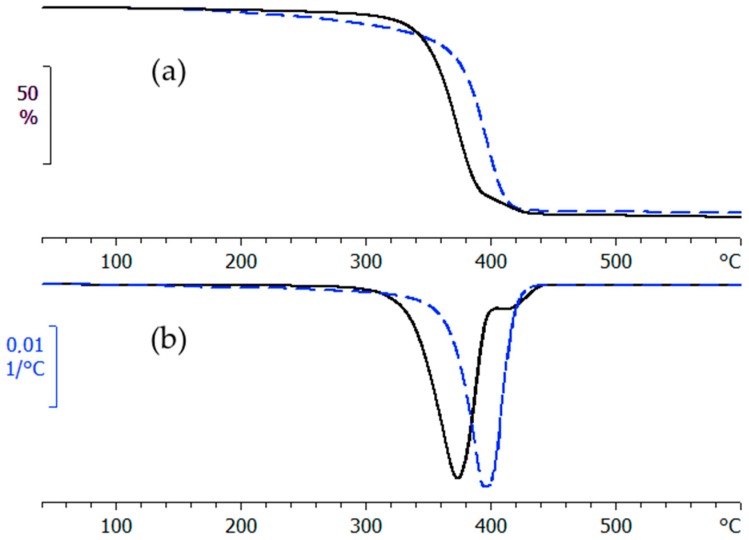
Comparative (**a**) TGA and (**b**) DTGA curves for PEI800 (dashed line, blue) and PEI25000 (full line, black) at the heating rate of 10 °C/min.

Thermal degradation curves for the hyperbranched polymers, PEI800 and PEI25000, with different molecular weights, are shown in Figure 3. The hyperbranched PEI25000 is thermally more stable than PEI800 up to about 320 °C, as measured by the value of the temperature corresponding to 5% weight loss, *T*_D5__%_, given in Table 1. The corresponding values for the HBPEI with an MW of 2000 g/mol, PEI2000, found in our earlier work [69], are also included in Table 1, and fall between those for PEI800 and PEI25000, but are much closer to the latter. At higher temperatures, we observe a sharp increase in the percentage weight loss, characterised by the onset temperature and by a maximum in the differential DTGA curves, both of which occur for PEI25000 at temperatures between 15 and 25 °C below the corresponding temperatures for PEI800. In this respect, and in contrast to the 5% weight loss, the PEI800 sample is much more thermally stable than PEI25000. If we include the results for the PEI2000 sample, both the onset and DTGA peak temperatures are higher than those for PEI800, implying that there is no simple relationship between MW and degradation, but suggesting that the PEI2000 sample is generally the most thermally stable.

When the HBPs are mixed with the epoxy resin, though, these differences in degradation behaviour are not apparent. In this context, it should be noted that the ELP mixtures cure during the TGA measurement, and it is the cured sample which degrades. This has been confirmed by repeating the TGA experiment for samples previously cured isothermally, and essentially the same degradation behaviour is observed as for the ELP samples. The values given in Table 1 show, for example, that the DTGA peak temperatures for the ELP mixtures all fall within the rather narrow ranges of 338 ± 5 °C for a 2 °C/min heating rate and 369 ± 5 °C for a 10 °C/min heating rate, with similar results (not included in Table 1) for the previously cured samples. We conclude that there is no significant or systematic effect of MW on the degradation behaviour of the cured systems.

### 2.2. Differential Scanning Calorimetry (DSC) Results

The results of the calorimetric analysis carried out by DSC and TOPEM a stochastic temperature modulated DSC technique, are summarised in Table 2. It can be seen that the glass transition temperatures, *T*_g0_, for the hyperbranched polymers PEI800 and PEI25000, for example at 2 °C/min, are −62 °C and −53 °C, respectively, with the temperature increasing with the MW of the HBPEI. This is consistent with the earlier results for PEI2000, shown in Table 2 [69], with an intermediate MW, for which *T*_g0_ at the same heating rate of 2 °C/min was −55 °C, which is much closer to the value for PEI25000 than to that for PEI800. This systematic increase in *T*_g0_ with MW of the HBPEI could be due to an increased mobility as the MW decreases, as a consequence of a reduced interaction between the side chains. Later (Figure 11), it will be seen that this same effect is observed in the glass transition of these HBPEIs, determined as the α-relaxation by DRS.

The average glass transition temperature for the unreacted epoxy resin is −17 °C. The glass transition temperatures, *T*_g0_, of the mixtures of the HBPEIs with the epoxy resin, ELP800 and ELP25000, before curing, give values between −17 and −22 °C, which is slightly less than that for the epoxy resin but within the same range as that found earlier for ELP2000, shown in Table 2, implying that there is no significant effect of the MW of HBPEI on *T*_g0_ of the ELP mixtures.

**Figure 4:**
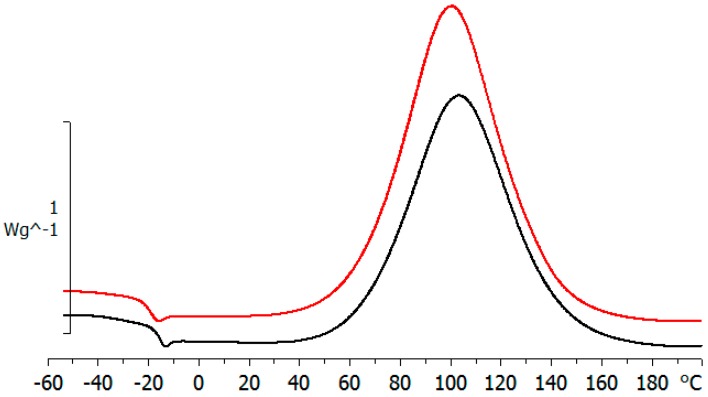
DSC scan for ELP800 (red line, upper curve) and ELP25000 (black line, lower curve) at 10 °C/min.

The crosslinking reaction, monitored by conventional DSC, shows that the cure of the ELP800 system starts before that of ELP25000, with the peak temperature, *T*_p_, of the exotherm being lower for ELP800 than for ELP25000, as can be seen from the values in Table 2 and illustrated in Figure 4. Also, the results in Table 2 show that the heat of the reaction for ELP800 is greater than that for ELP25000, while the average value for ELP2000 lies between these two values. All these results indicate that the epoxy HBPEI mixtures become more reactive as the MW decreases; this is related to the structural feature of the HBPEI component whereby there is a greater number of H equivalents per gram of HBPEI the lower the MW is. In the second DSC scan, at 10 °C/min, no residual reaction is observed. The glass transition temperature of the fully cured sample, *T*_g∞_, has been determined for both systems and is included in Table 2. It can be seen that *T*_g∞_ increases slightly with MW when comparing ELP25000 with ELP800, but that *T*_g∞_ for ELP2000 does not follow this trend; we believe that this is due to the uncertainty in the measurement of *T*_g∞_ for ELP2000, for which the glass transition is extremely broad and poorly defined. In fact, this discrepancy is not seen in the results for either TOPEM or the isothermally cured films listed in Table 2, where the slight increase in *T*_g∞_ with MW is confirmed.

**Figure 5:**
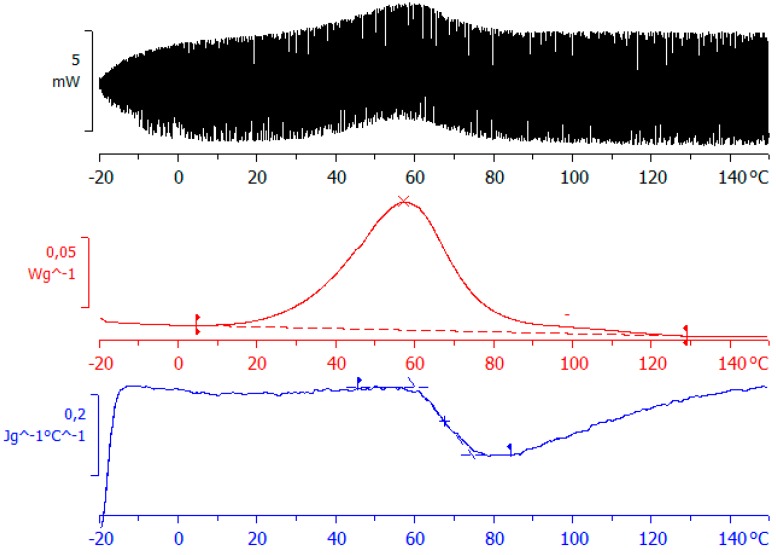
TOPEM thermogram for ELP25000 at an underlying heating rate of 0.5 °C/min: top diagram shows the stochastically modulated heat flow; middle diagram shows the total heat flow; bottom diagram shows the “quasi-static” specific heat capacity, *c*_p0_.

A more complete picture of the curing process can be obtained by the use of TOPEM. A typical result, obtained for ELP25000, is shown in Figure 5. The upper curve shows the heat flow that results from the stochastically generated pulses of temperature change, from which an underlying or total heat flow curve can be derived, as shown in the middle diagram, equivalent to the heat flow curve that would be obtained by conventional DSC. The heats of the reaction for the two ELP samples are given in Table 2, and are similar to those from conventional DSC; in particular, they show the same trend of an increasing heat of reaction as the MW of the HBPEI decreases. The stochastically modulated heat flow curve in the upper diagram can be deconvoluted to give a frequency dependent specific heat capacity, the “reversing” component of the modulations, which in the limit, gives the so-called “quasi-static” specific heat capacity, *c*_p0_, shown as the lower curve in Figure 5, and which has been shown to be equivalent to a limiting frequency of approximately 4 mHz [75]. This curve displays a sigmoidal reduction in *c*_p0_, indicative of vitrification taking place during cure at the lowest heating rate of 0.5 °C/min [76,77,78]. Both systems, ELP800 and ELP25000, show a very similar vitrification behaviour in non-isothermal cure at 0.5 °C/min, with vitrification occurring around 67 °C (Figure 5, Table 2), similar to the vitrification of ELP2000 reported earlier [69]. This indicates that the vitrification behaviour of these systems is not influenced by the effect of the MW of the HBPEI.

### 2.3. Dielectric Relaxation Spectroscopy (DRS)

#### 2.3.1. PEI800 and PEI25000 Systems

**Figure 6:**
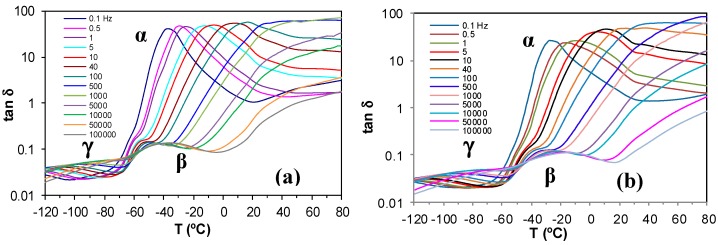
Dielectric relaxation curves for (**a**) PEI800 and (**b**) PEI25000. Heating rate: 0.5 °C/min.

The dielectric relaxation curves for the PEI800 and PEI25000 samples are shown in Figure 6, in the representation of tan δ as a function of temperature, for the heating rate of 0.5 °C/min and over a range of frequencies, *f*. Two dipolar secondary relaxations are observed: the γ-relaxation and the β-relaxation. The γ-relaxation, which occurs at temperatures below −70 °C and over a wide frequency range, can be attributed to the mobility of the end groups (NH_2_) present on the branches. The dipolar β-relaxation can be observed in the temperature range between about −70 °C and −20 °C for PEI800, becoming better defined for frequencies greater than 10 Hz, while this relaxation for PEI25000 appears at higher temperatures, in the range from −50 °C to 0 °C, again becoming more clearly defined at higher frequencies. At temperatures higher than that of the β-relaxation, a much stronger peak is observed. This peak corresponds to the dipolar α-relaxation, associated with the glass transition of the material.

**Figure 7:**
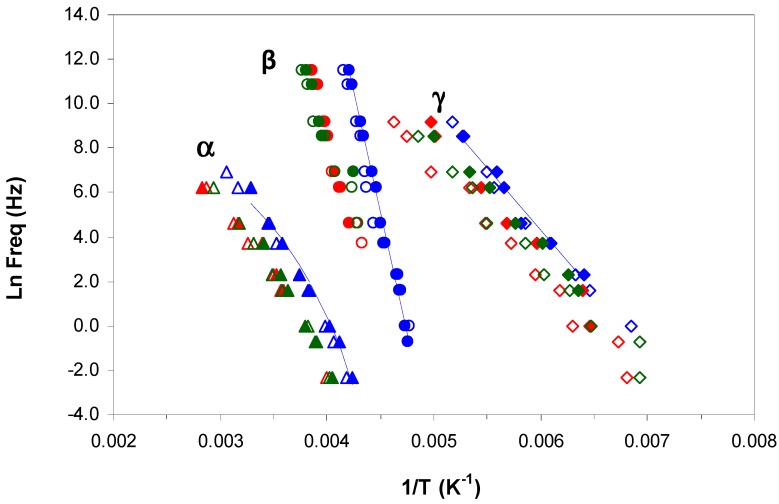
Relaxation map for PEI800 (blue), PEI2000 (green), and PEI25000 (red) at heating rates of 0.5 °C/min (filled symbols) and 2 °C/min (open symbols).

The results for all these relaxations can be combined to give a relaxation map for the HBPEI at different heating rates, as shown in Figure 7. It can be seen that, for all the relaxations, the heating rate has practically no influence on the activation energy, while the temperature of the relaxation peaks increases with the MW of the HBPEI; the same effect was observed above for the glass transition temperature of the HBPEIs determined by DSC (Table 2). Each of these relaxations is now considered in turn.

For the γ-relaxation, the dependence of ln(*f*) on the reciprocal of the temperature is linear, following an Arrhenius dependence. The average value of the activation energy of this relaxation is 46 ± 2 kJ/mol for PEI800 and 44 ± 2 kJ/mol for PEI25000, while for PEI2000, the average value was earlier found to be 42 ± 2 kJ/mol [69], thus being independent of MW as might be expected. However, this result also suggests that these terminal groups all exist in the same environment. These results all fall within the range of values for most other hyperbranched polymers, from 22 to 45 kJ/mol, as compiled from the literature [69], though much higher values have been reported for hyperbranched polyesters [74,79,80].

For the β-relaxation, the ln(*f*) vs. *T*^−1^ representation shown in Figure 7 is linear, and the average activation energies of 168 ± 20 kJ/mol for PEI800 and 170 ± 20 kJ/mol for PEI25000 are essentially the same (Table 3). The β-relaxation for PEI2000 was earlier observed [69] to be in the temperature range between −50 and −10 °C, but the overlap with the α-relaxation made it appear as a shoulder rather than a peak over almost the whole frequency range; the average activation energy of 92.7 kJ/mol was obtained from an analysis of the onset temperatures rather than the unavailable peak temperatures, and in the light of the present results, we prefer to ignore this earlier value. For this relaxation, therefore, which can be attributed to the mobility of the branches, we conclude that the MW of the HBPEI influences the temperature range in which the relaxation appears, being displaced to higher temperatures the higher the MW is, but without any change in the activation energy.

For the α-relaxation, and comparing it at the same frequency, the temperature at which this relaxation occurs depends on the molecular weight of the HBPEI. For example, at the lowest frequency of 0.1 Hz in Figure 6, the α-relaxation peak for PEI800 appears at about −37 °C, while that for PEI25000 appears at about −28 °C. This difference of about 10 °C is in agreement with the difference in the *T*_g_ values obtained by DSC at 2 °C/min and shown in Table 2, −62 °C and −53 °C, respectively, while the actual values are of course much higher for the α-relaxation by DRS as the frequency is higher than the equivalent frequency for the DSC experiments. The peak temperature at 0.1 Hz for PEI2000 [69] is at about −27 °C, which is very similar to the value of −28 °C found for PEI25000, thus showing the same dependence on MW as for the *T*_g_ values obtained by DSC at 2 °C/min.

The α-relaxation peak for the HBPEIs studied here is an asymmetric peak in which the presence of a shoulder on the high temperature side can be clearly observed in Figure 6. The presence of this shoulder was also evident for PEI2000 [69], and seems to suggest the existence of a relaxation peak, overlapping the dipolar α-relaxation and associated with ionic charge trapped in the HBPEI [81]. The effect of the MW of the HBPEI on this ionic relaxation can be seen here: with increasing MW of the HBPEI, this shoulder corresponding to the ionic relaxation becomes more pronounced and is clearly evident over a wider frequency range.

In contrast to the sub-*T*_g_ γ- and β-relaxations, the relationship between ln(*f*) and the reciprocal temperature for the α-relaxation is non-linear, following a Vogel-Fulcher-Tammann (VFT) dependence, as seen in Figure 7. The parameters of this equation, (ln(*f*) = *A* − *B*/(*T* − *T*_0_)), are normally determined by a least squares fit of the VFT equation to the data, but the range of temperatures and frequencies used experimentally here are insufficient to obtain reliable values for these parameters. Instead of fitting the VFT equation, therefore, we calculate an apparent activation energy, *E*_app_, from the slope of a best fit straight line to the data for the α-relaxation. The values obtained for this apparent activation energy are show in Table 3 for the different heating rates. The average activation energy for the α-relaxation of PEI800 is 69 kJ/mol, while that for PEI25000 is 59 kJ/mol, compared with 71 kJ/mol for PEI2000 [69]. The location of the data for PEI2000 on the relaxation map of Figure 7 is much closer to PEI25000 than to PEI800, suggesting that the mechanism of the α-relaxation is the same for all the HBPEIs, but that the relaxation is shifted to somewhat lower temperatures for the lowest MW.

In the same way as for PEI2000 [69], the β- and γ-relaxations of the PEI800 and PEI25000 are independent of the α-relaxation; in particular, the β-relaxation does not present the appearance of a Johari-Goldstein (JG) relaxation, according to its common classification [82], in contrast to what is observed for the pure epoxy resin [69]. For some other hyperbranched polymers, such as polyglycerols [83] and polyester amides [84], the β-relaxation presents JG behaviour, whereas in others, for example, polyesters [80], it does not.

#### 2.3.2. Epoxy Resin

The dielectric relaxation behaviour of the epoxy resin used in this work has been widely studied in previous work [69]. Two secondary relaxations are observed, γ and β, whose activation energies are 28 kJ/mol and 80 kJ/mol, respectively, which are lower than those for all three HBPEIs. The α-relaxation for the epoxy, on the other hand, has an apparent activation energy of 306 kJ/mol, which is much higher than that for the HBPEIs. These values for the activations energies are included in Table 3 for comparison with the other systems.

#### 2.3.3. ELP800 and ELP25000 Uncured Systems

**Figure 8:**
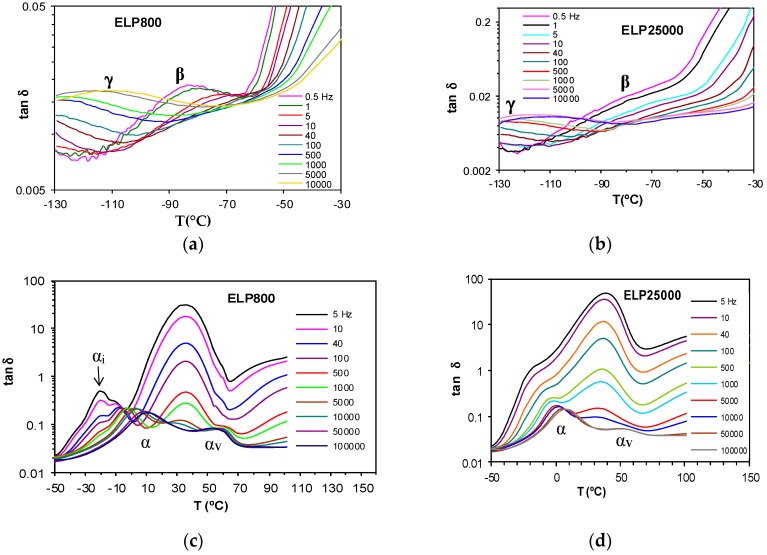
Dielectric relaxation curves for tan δ for the uncured ELP samples at 0.5 °C/min. Upper figures from −130 °C to −30 °C: (**a**) ELP800 and (**b**) ELP25000; lower figures from −50 °C to 150 °C: (**c**) ELP800 and (**d**) ELP25000.

The uncured systems ELP800 and ELP25000, consisting of the mixtures of HBPEI and epoxy resin before effecting the curing reaction, show two secondary relaxations, γ and β, at temperatures below −30 °C, as shown in Figure 8a,b for the heating rate of 0.5 °C/min. The appearance of the γ-relaxation is very similar for both systems, whereas the β-relaxation for the ELP800 system is clearly visible at low frequencies, while it appears only as a shoulder for the ELP25000 system. The average activation energies for the γ-relaxation at the two different heating rates are 29.1 ± 2 kJ/mol for ELP800 and 33.5 ± 2 kJ/mol for ELP25000, and are included in Table 3. The activation energies for the γ-relaxation for these ELP systems, and also including the average value of 31.5 kJ/mol for ELP2000 from earlier work [69], lie between the average values for the individual components, HBPEI (45.8 kJ/mol) and epoxy (27.7 kJ/mol). In fact, there is a trend of increasing activation energy as the MW of the HBPEI increases.

For the β-relaxation of these ELP systems, only the system ELP800 can be analysed as ELP25000 shows only a very slight shoulder instead of a peak. The average activation energy for the ELP800 system of 72 kJ/mol, which is close to the value for the epoxy resin (80 kJ/mol), can be compared with that found earlier [69] for the ELP2000 uncured system, namely 81 ± 7 kJ/mol. The activation energy of this relaxation increases with the MW of the HBPEI.

The dielectric spectra of these systems at higher temperatures are shown in Figure 8c,d for ELP800 and ELP25000, respectively. For the ELP800 system, shown in Figure 8c, there are as many as four different peaks (or shoulders) in the complete scan, corresponding to distinct relaxation processes. The lowest temperature relaxation, occurring at around −20 °C, displays a peak denoted α_i_, or, for frequencies greater than 40 Hz, a shoulder superimposed on the next higher relaxation, which is denoted α. With increasing frequency, this α_i_ shoulder remains at a constant temperature but reduces in intensity, and for frequencies greater than 1 kHz, the α_i_-relaxation is no longer visible. While the α_i_-relaxation reduces in intensity with increasing frequency, the α-relaxation increases, and also shifts to higher temperatures. Consequent upon these observations, the α_i_-relaxation, whose position does not change with frequency, is considered to be an ionic peak, while the α-relaxation, for which the peak temperature increases with increasing frequency, is considered to be dipolar and to correspond to the glass transition.

The α_i_-relaxation could correspond to the glass transition of the minor component (HBPEI), which would be acting as an impurity and would be the origin of the ionic relaxation peak. The ELP800 system is phase separated, as has been shown in Figure 2, with the HBPEI as a minor component surrounded by epoxy resin. The DRS results indicate a much higher glass transition temperature for the HBPEI in the ELP mixture than in the neat state, and with a behaviour more similar to an ionic process than a dipolar one, both of which may be as a consequence of changes in the molecular mobility of the HBPEI in the two-phase morphology. This would explain why only a single glass transition was observed in the uncured ELP800 sample by DSC, which is not sensitive enough to detect these two overlapping transitions. On the other hand, the α-relaxation is attributed to the glass transition of the major component, the epoxy resin. This behaviour has also been observed in the study of the mixture of hyperbranched Lupasol PR8515 (PEI2000), with the epoxy resin (ELP2000) [69].

The higher MW ELP25000 sample shows a dielectric spectrum slightly different in this temperature range, as seen in Figure 8d. Here, there is no evidence of a separate α_i_-relaxation, which is consistent with the observation by optical microscopy, shown in Figure 2, that this uncured mixture is not phase-separated. The α-relaxation first appears as a shoulder on a broad peak centred at about 40 °C, and is then transformed into a well-defined peak as the frequency increases (*f* > 100 Hz). The position of this α-relaxation peak can be seen to increase with frequency.

At temperatures above the α-relaxation, in other words, above the glass transition temperature, a broad peak is observed in both samples, ELP800 and ELP25000. This peak is slightly sharper in the ELP800 sample that in ELP25000 and corresponds to the curing reaction of the ELP system, which begins when the system has sufficient mobility when it is above *T*_g_. In the curing reaction, there is a maximum in tan δ, occurring at a temperature which is not dependent on the frequency, and which is associated with the minimum viscosity of the system. 

This peak extends from about 0 °C to 60 °C and is ionic in origin. With continued heating, the curing reaction progresses and at temperatures above 60 °C, and for the higher frequencies, another relaxation, denoted α_v_, becomes visible. The peak of this relaxation shifts to slightly higher temperatures as the frequency decreases, is dipolar in origin, and is attributed to the vitrification of the system [69]. For frequencies below 1 kHz, the relaxation corresponding to vitrification appears as a shoulder on the ionic peak of curing, which becomes more evident as the frequency increases. The vitrification of these samples can also be observed using the technique TOPEM, where a rather abrupt change occurs in the so-called “quasi-static” specific heat capacity, *c*_p0_, corresponding to the vitrification of the sample, as shown by Fraga et al. [78,85] and by Van Mele and co-workers [76], and which can be seen in the lower diagram of Figure 5. The temperature at which vitrification takes place is measured as the midpoint of the sigmoidal change in *c*_p0_, as shown in Figure 5.

**Figure 9:**
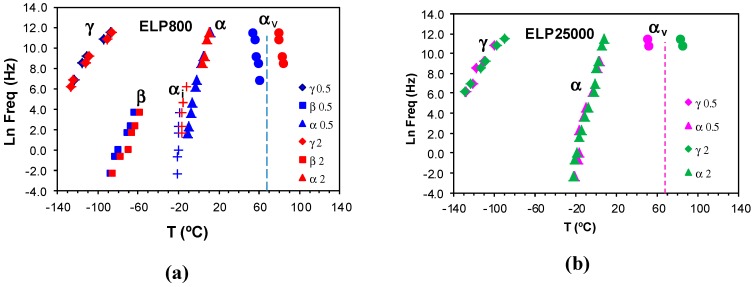
Temperatures of the relaxations peaks for the uncured (**a**) ELP800 and (**b**) ELP2500 samples at different heating rates: 0.5 °C/min (ELP800, blue symbols; ELP25000, pink symbols) and 2 °C /min (ELP800, red symbols; ELP25000, green symbols). The vertical dashed line shows the vitrification temperature obtained by TOPEM from *c*_p0_ at 0.5 °C/min.

The peak temperatures are determined for the various relaxations in the ELP800 and ELP25000 systems, at the heating rates of 0.5 and 2 °C/min, and their variation with frequency is plotted in Figure 9. The dashed line corresponds to the vitrification temperature determined by TOPEM from the quasi-static specific heat capacity, *c*_p0_, for which it has been demonstrated that the corresponding frequency is about 4 mHz [75]. As can be seen, the γ-, β-, and α-relaxations show no significant dependence on the scan rate and, in addition, the α_i_-relaxation appears only for the ELP800 system, for reasons explained above. In contrast, the α_v_-relaxation is significantly affected by the heating rate, occurring at higher temperatures the faster the heating rate is, in agreement with the TOPEM results [77,78]. At a constant frequency, the vitrification peak α_v_ is dependent on the MW of the HBPEI, decreasing in temperature as the MW increases. Thus, at the heating rate of 0.5 °C/min and at the highest frequency, for example, α_v_ is observed at approximately 57 °C in ELP800, while in ELP2000 [69], it is observed at 55 °C and in ELP25000 at 50 °C. At the same heating rate, the values obtained by TOPEM are 67 °C in ELP800, 65 °C in ELP2000 [69], and 68 °C in ELP25000. These should represent the temperatures to which the data for the α_v_-relaxations by DRS extrapolate for a frequency of 4 mHz (ln*f* = −5.5), which does not appear unreasonable in Figure 9.

**Figure 10:**
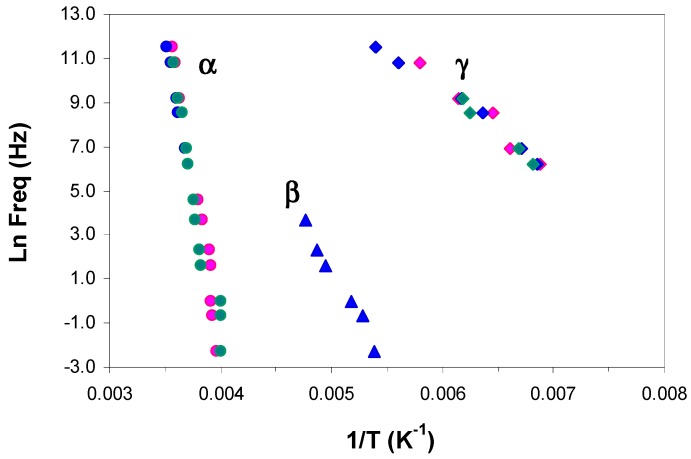
Relaxation map for ELP800 (blue), ELP2000 (green), and ELP25000 (pink) uncured systems at 0.5 °C/min: α-relaxation, circles; β-relaxation, triangles; γ-relaxation, rhombus.

The relaxation map for the uncured ELP800 and ELP 25000 systems at 0.5 °C/min is shown in Figure 10. As can be seen, the γ-relaxation temperatures are essentially independent of the molecular weight of HBPEI, and this is reflected in the activation energies collected in Table 3: the average values are 30 kJ/mol and 36 kJ/mol for ELP800 and ELP 25000, respectively. The β-relaxation is not shown for either the ELP25000 or the ELP2000 systems, as this relaxation appears only as a shoulder rather than a peak for these ELP mixtures. As regards the α-relaxation in the ELP systems, it can be seen that there is essentially no effect of the MW, and the earlier results for ELP2000 [69] also fall approximately on the same curve. The average apparent activation energies for the three ELP systems are 276 kJ/mol, 284 kJ/mol, and 322 kJ/mol in order of increasing MW, with the small but systematic trend possibly being significant. On the other hand, these activation energies are all much higher than those for the corresponding HBPEIs, which range from 60 to 78 kJ/mol for the three systems, but are close to that for the epoxy resin, 306 kJ/mol.

**Figure 11:**
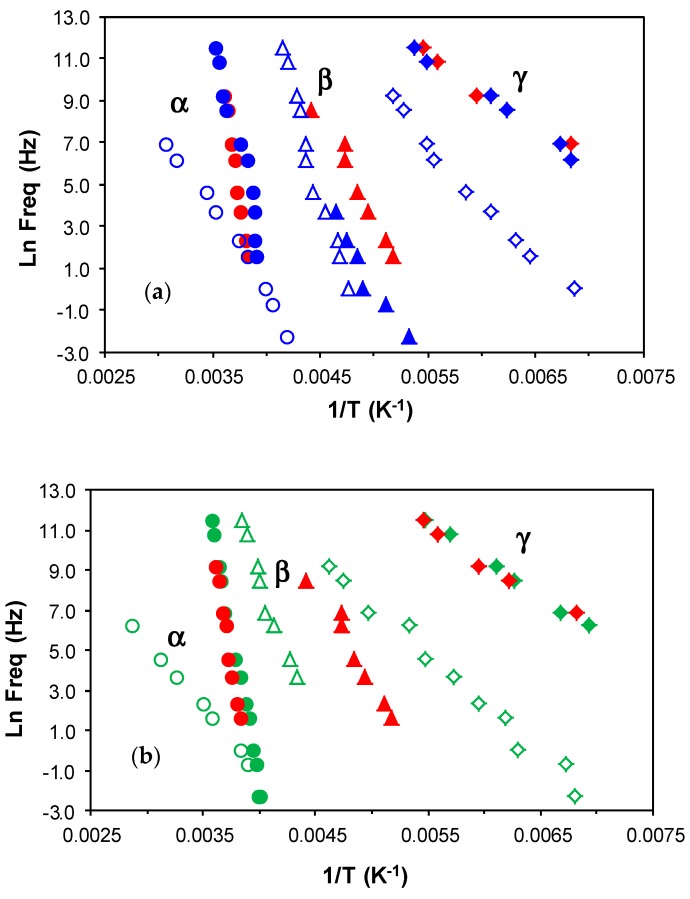
Relaxation map comparing epoxy (red) with HBPEIs (open symbols) and uncured ELP mixtures (filled symbols) of different MWs: (**a**) PEI800, ELP800, blue symbols; (**b**) PEI25000, ELP25000, green symbols. α-relaxation (circles); β-relaxation (triangles); γ-relaxation (rhombus). Heating rate 2 °C/min.

Figure 11a,b compare the relaxation behaviour of epoxy with the HBPEIs and uncured ELP mixtures of MW 800 and 25000, respectively. As can be seen, the α- and γ-relaxations for the ELP systems coincide very closely with those for the epoxy resin, and for this reason are considered to be due to the contribution of the major component, i.e., the epoxy resin. This is reflected in their activation energies, given in Table 3. The same correspondence was also noted earlier for ELP2000, and was illustrated in Figure 9 of reference [69]. In contrast, the HBPEIs show a noticeably different behaviour for these two relaxations.

For the β-relaxation, however, while there is again a noticeable difference between the behaviours of the epoxy and the HBPEIs, the correspondence between epoxy and ELP systems is not evident. The reason for this is, at least in part, the overlap of the α- and β-relaxations. For the ELP2000 and ELP25000 systems, the β-relaxation appears only as a shoulder on the α-relaxation, while for ELP800, a distinct peak appears only for the lowest frequencies and becomes increasingly combined with the α-relaxation as the frequency increases. 

#### 2.3.4. ELP800 and ELP25000 Fully Cured Systems

**Figure 12:**
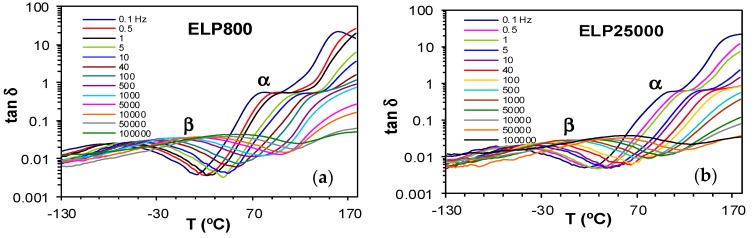
Dielectric relaxation curves at 2 °C/min for the fully cured systems: (**a**) ELP800; (**b**) ELP25000.

Dielectric relaxation curves for the fully cured ELP800 and ELP25000 systems are shown in Figure 12. The γ-relaxation present in the uncured ELP mixtures is no longer evident in these fully cured samples, as it was associated with end groups which are consumed in the crosslinking reaction. Instead, only one secondary relaxation, the β-relaxation, is observed, which is much better defined over a wide frequency and temperature range than it was for the uncured ELP systems. This relaxation shows a peak in tan δ at a temperature which increases slightly with the MW of the HBPEI, and is related to the mobility of segments of the branches between crosslinks. The α-relaxation for these fully cured systems becomes visible as a shoulder at temperatures above about 70 °C, depending on the frequency and on the MW, with the relaxation occurring at higher temperatures for the ELP25000 system. Very similar results were also found for the cured ELP2000 system [69]. This is consistent with the results obtained by DSC presented above, where a higher glass transition temperature was found for the ELP system with the higher MW HBPEI, as shown in Table 2. This α-relaxation appears only as a shoulder as it is overlapped with conductivity effects in the DRS curves.

**Figure 13:**
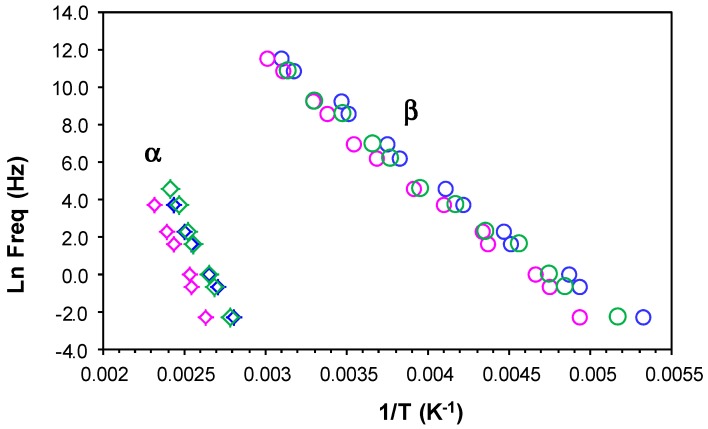
Relaxation map for the fully cured ELP800 (blue), ELP25000 (pink), and ELP2000 (green) systems obtained by DRS at 2 °C/min.

The relaxation map for the fully cured ELP800 and ELP25000 systems is shown in Figure 13. The representation of ln(*f*) vs. *T*^−1^ for the β-relaxation follows an Arrhenius dependence. The slope of the best-fit line to the experimental data gives the activation energy for this relaxation as 53.1 kJ/mol for ELP800 and 57.6 kJ/mol for ELP25000 (Table 4), very close to the value of 53.8 kJ/mol found earlier for fully cured ELP2000 at the same heating rate of 2 °C/min [69], and similar to the value of 63.4 kJ/mol reported by Tombari et al. [86,87] for DGEBA cured with a diamine.

For the α-relaxation, there is a shoulder rather than a peak, which makes the analysis less precise. Instead of a peak temperature, we use an onset temperature, determined as the intersection of the inflectional tangents before and after the shoulder, to define the temperature of the α-relaxation for each frequency. The parameters of the VFT equation are normally determined by a least squares fit of the VFT equation to the data, but for the results presented in Figure 13, there is an insufficient range of temperature and frequency to obtain reliable values for these parameters. Instead of fitting the VFT equation, we calculate an apparent activation energy from the average slope of a best fit straight to the data for the α-relaxation, which gives values of 128 kJ/mol and 159 kJ/mol for ELP800 and ELP25000, respectively, for the heating rate of 2 °C/min. These values should be compared with the value of 158 kJ/mol obtained in earlier work for fully cured ELP2000 [69]. Although it might appear that the apparent activation energy is greater for the systems based upon the higher MW HBPEIs, the uncertainties associated with the evaluation of these activation energies are too large to conclude this.

### 2.4. Dynamic Mechanical Analysis (DMA)

#### 2.4.1. HBPEIs and Uncured ELP Mixtures

**Figure 14:**
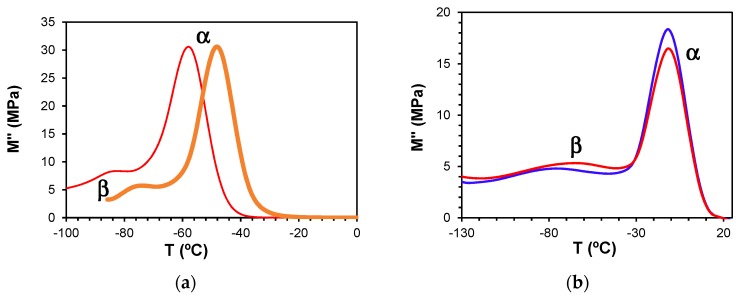
Loss modulus obtained by DMA at the heating rate of 2 °C/min for the systems studied: (**a**) PEI800 (red) and PEI25000 (brown) at 0.1 Hz; (**b**) uncured ELP800 (blue) and uncured ELP25000 (red) at 1 Hz.

Dynamic mechanical measurements of the pure components, namely the HBPEIs, and of the ELP systems, manifest a single secondary relaxation, the β-relaxation, unlike the two secondary relaxations γ and β observed by dielectric spectroscopy, as well as the α-relaxation, as shown in Figure 14. Likewise, although not shown here, the pure epoxy also displays only a single secondary relaxation [69]. For the pure HBPEI, there is a clear shift of both the α- and β-relaxation peaks to a higher temperature as the MW of the hyperbranched polymer increases (Figure 14a).

At a frequency of 0.1 Hz, the β-relaxation presents a maximum at −84 °C for PEI800 that increases to −82 °C for PEI2000 [69] and to −76 °C for PEI25000. The α-relaxation is observed at −58 °C for PEI800, while for PEI2000, it is observed at −50 °C [69] and for PEI25000 at −48 °C, at the same frequency. These DMA results for the HBPEIs appear to be in agreement with the DRS results shown in Figure 6: the relaxation peaks move to a higher temperature with increasing MW. For the uncured ELP mixtures, there is a similar shift only for the β-relaxation: at the frequency of 1 Hz, the β-relaxation is observed at −80 °C for ELP800, at −71 °C for ELP2000 [69], and at −64 °C for ELP25000. In contrast, the temperature of the α-relaxation remains practically constant with MW, at approximately −11 °C (Figure 14b), the same temperature at which the peak was earlier found for ELP2000 (Figure 14 of Reference [69]); this is to be expected, as it was observed from the DRS results that it is the epoxy resin which dominates this relaxation in these mixtures.

**Figure 15:**
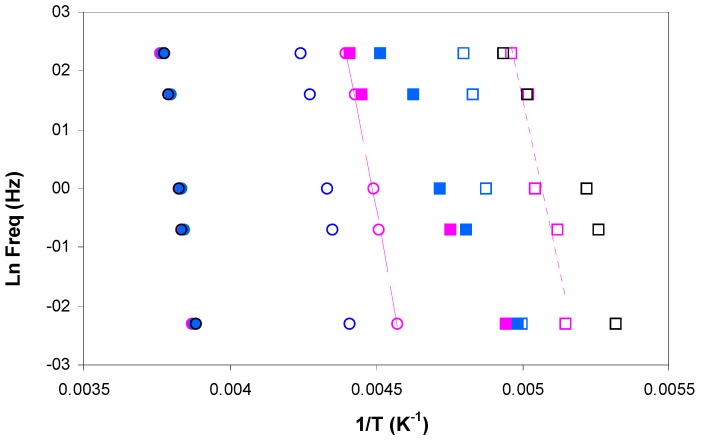
Relaxation map showing α-relaxation (circles) and β-relaxation (squares) obtained by DMA measurements at 2 °C/min. Open symbols: epoxy (black), PEI800 (pink), PEI25000 (blue). Filled symbols: uncured ELP800 (pink), uncured ELP25000 (blue).

The DMA relaxation map for all the samples studied, at the heating rate of 2 °C/min, is shown in Figure 15. The activation energies for the β- and α-relaxations obtained from these plots are included in Table 5. For the β-relaxation, the peak temperature increases with MW, but the activation energies for PEI800 (194 kJ/mol) and for PEI25000 (192 kJ/mol) are very similar, suggesting that the molecular mechanism is not influenced by the MW, possibly indicative of a similar structure in which the side groups have the same ease of movement. Interestingly, the β-relaxation for PEI2000 [69] appears only as a shoulder, but occurs in a temperature range intermediate between PEI800 and PEI25000. Similar observations were made above for the DRS experiments: the temperature of the β-relaxation increases with MW (Figure 7); the relaxation for PEI2000 appeared only as a shoulder; and the activation energies were essentially independent of MW, though the values obtained (see Table 3) by DRS at 2 °C/min, 154 kJ/mol and 169 kJ/mol for PEI800 and PEI25000, respectively, are slightly lower than those found by DMA.

For the uncured ELP systems, the activation energy for the β-relaxation is very much lower than for the HBPEI polymers, which is the same observation that was made above in respect of the DRS results. A small increase with MW, from 69.1 kJ/mol for ELP800 to 84.2 kJ/mol for ELP25000, is consistent with an intermediate value of 79.0 kJ/mol for ELP2000 from earlier work [69], though this dependence on MW may not be significant. The activation energy obtained by DMA for the β-relaxation for all these ELP uncured mixtures remains close to that found for the epoxy resin by DMA, namely 89.0 kJ/mol, again in agreement with the results obtained by DRS experiments, but occurs in a temperature interval significantly different from that for the epoxy resin. This is in direct contrast to the comparison of the α-relaxation temperatures, considered immediately below.

As regards the α-relaxation, it can be seen in Figure 15 that the ELP800 and ELP25000 systems both superpose on the results for the epoxy resin, which is reflected in similar values for the apparent activation energy given in Table 5; the earlier results obtained for ELP2000 [69] are also included in this Table, and likewise superpose on the results for the epoxy resin. The activation energies for the uncured ELP mixtures are significantly higher than those obtained for the HBPEIs (224 kJ/mol and 234 kJ/mol), consistent with earlier results for PEI2000 [69] and with the results obtained by DRS (Figure 11), though the difference between HBPEI and ELP/epoxy observed by DRS is much greater. Comparing the *E*_app_ values obtained by both measurement techniques, it can be seen that the values obtained for the ELP systems by DMA, with an average of about 360 kJ/mol, are higher than those obtained by DRS, with an average of about 300 kJ/mol (Table 3).

#### 2.4.2. ELP800 and ELP25000 Fully Cured Systems

**Figure 16:**
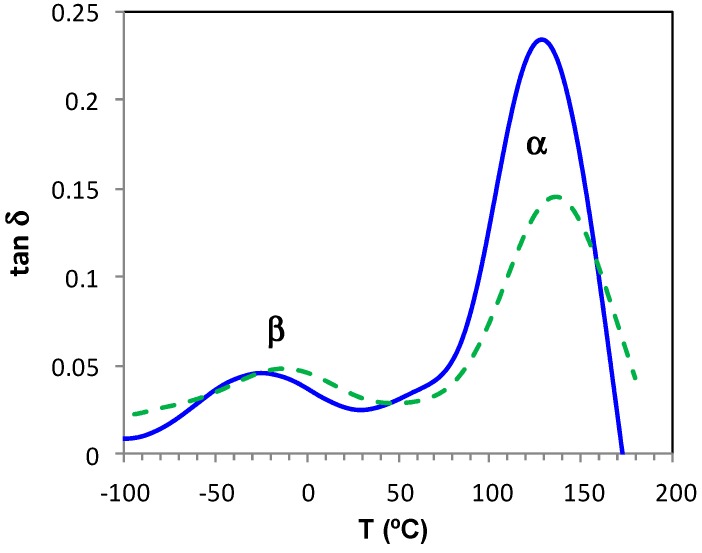
DMA curves for the fully cured ELP800 (full line) and ELP25000 (dashed line), systems at 5 Hz and 2 °C/min.

**Figure 17:**
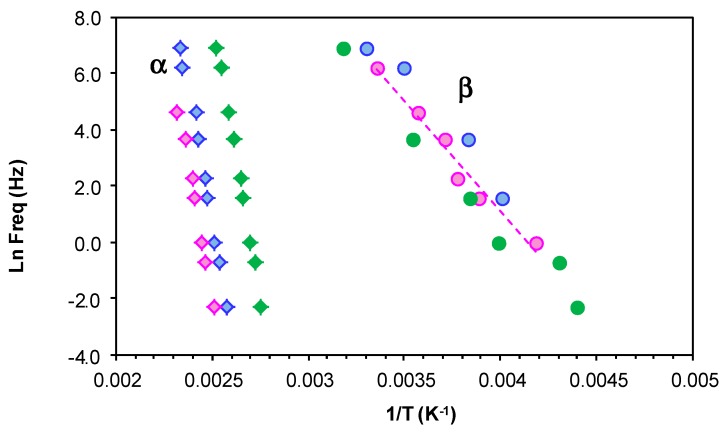
Relaxation map for the fully cured ELP800 (blue), ELP2000 (green), and ELP25000 (pink) systems obtained by DMA at 2 °C/min; α-relaxation, rhombus; β-relaxation, circles.

The dynamic mechanical measurements for the fully cured ELP800 and ELP25000 systems, at the frequency of 5 Hz and with a heating rate of 2 °C/min, can be seen in Figure 16, where two relaxations are clearly observed: a β-relaxation at temperatures below 50 °C and an α-relaxation, very well defined, in the range between 50 °C and 200 °C. The intensity of the β-relaxation peak is rather low, and is essentially the same in both systems, while the intensity of the α-relaxation peak is significantly higher, and is greater for ELP800 than for ELP25000. The temperatures at which the maxima of these peaks occur are dependent on the MW of the HBPEI and on the frequency, as can be clearly seen in the relaxation map shown in Figure 17.

In Figure 17, it can be seen that the β-relaxations for all three fully cured ELP systems superpose approximately, with an average activation energy of 61.5 kJ/mol, with the scatter being a consequence of the uncertainty in defining the peak temperature for these rather shallow peaks. If anything, the β-relaxation for the ELP2000 system occurs at a higher temperature than for the other two systems, while maintaining the same activation energy. In a similar way, the α-relaxation displays a dependence in which the activation energy is again constant, but the location of the relaxation peak depends on MW, though again not in a systematic way: the α-relaxation for ELP2000 occurs in a temperature range less than that for the other systems of lower and higher MW. Furthermore, the representation of ln(*f*) vs. *T*^−1^ is clearly linear, so fitting a VFT equation to these data would not be appropriate. Instead, we determine an apparent activation energy for each sample, giving 319 kJ/mol and 332 kJ/mol for ELP800 and ELP25000, respectively, which are both very close to the value of 323 kJ/mol obtained earlier [69] for ELP2000, indicating that the mechanism of the mechanical α-relaxation is the same in all these systems, independent of the MW of the HBPEI. Thus, it seems that, while the α- and β-relaxations in these fully cured ELP systems have activation energies independent of the MW of the HBPEI, for the ELP2000 system of intermediate MW, they occur at temperatures lower and higher, respectively, than those of the other ELP fully cured systems. This may imply that the structure formed is somehow different in these systems, perhaps more compact for ELP800 and ELP25000 and more irregular for ELP2000. This converging of the α- and β-relaxations in fully cured ELP2000 may be associated with the overlap of these relaxations in the uncured ELP mixture, observed by both DRS and DMA, which prevented the determination of the peak temperatures of the β-relaxation in this system.

Finally, it is interesting to observe that the activation energy for the mechanical α-relaxation is significantly higher than the average of about 150 kJ/mol obtained by DRS (see Table 4), likewise being essentially independent of molecular weight.

## 3. Materials and Methods

### 3.1. Materials

The epoxy resin used (DER 331, Dow Chemical Company, Midland, MI, USA) is a commercial diglycidyl ether of bisphenol-A (DGEBA), with an epoxy equivalent in the range 182–192 g/eq and a viscosity in the range 11,000–14,000 mPa·s. The hyperbranched polymers used are polyethyleneimines (HBPEI) of different molecular weights, provided by the BASF Chemical Company (Florham Park, NJ, USA) with tradenames: Lupasol^®^ FG, referred to here as PEI800 and with a molecular weight of 800 g/mol and viscosity at 20 °C in the range 800–5000 mPa·s; and Lupasol^®^ WF, referred to here as PEI25000 and with a molecular weight of 25,000 g/mol and viscosity at 50 °C in the range 13,000–18,000 mPa·s. Before performing any experiments, the samples were previously preconditioned at 100 °C for 30 min in order to remove any moisture content in the samples. The chemical structure of the hyperbranched polymer was shown above in Figure 1. The characteristics of the HBPEI samples for the amine ratio, equivalent H mass, equivalent N mass, and degree of branching are listed in Table 6.

### 3.2. Preparation of the Samples

The epoxy-Lupasol samples (ELP) were prepared by mixing the pure components manually on a watch glass, followed by degassing in a vacuum chamber (Heraeus, model RVT360, Hanau, Germany) for 10 min at room temperature and 100 Pa pressure. The mixtures are denoted: ELP800, with a composition of 84.7 wt % epoxy and 15.3 wt % Lupasol^®^ FG; and ELP25000, with a composition of 82.7 wt % epoxy and 17.3 wt % Lupasol^®^ WF. Immediately after mixing and degassing, a proportion of this sample was taken in order to perform the corresponding thermal analytical experiments and to observe their morphology under the optical microscope.

A cured plaque of the ELP mixture, with a thickness of 1.3 mm and lateral dimensions of 25 mm square, appropriate for the dielectric spectroscopy and dynamic-mechanical analysis, was prepared by isothermally curing in a Teflon mould, placed in a thermostatically controlled oven at 50 °C for 3 h, followed by a post cure at 140 °C for a further 3 h.

### 3.3. Experimental Techniques

A Leica DME polarising transmission optical microscope, equipped with a digital camera system, was used for observation of the morphology of the ELP system.

Thermogravimetric analysis (TGA), which measures the weight loss of the sample during a prescribed thermal history, was performed in a Mettler-Toledo TGA/DSC1 equipped with a sample robot and Huber cryostat (precision ± 0.1 °C, Huber, Offenburg, Germany). The TGA/DSC was calibrated using indium with a dry air flow of 200 mL/min and the experiments were performed with a dry nitrogen flow of 200 mL/min at heating rates of 2 °C/min and 10 °C/min over a temperature range from 40 °C to 600 °C.

The curing reactions were studied using a Mettler-Toledo DSC 821e differential scanning calorimeter (DSC) equipped with a sample robot and Haake EK90/MT intracooler (Thermo Haake, Karlsruhe, Germany). Additionally, a stochastic temperature modulated DSC technique, TOPEM, incorporated in the Mettler-Toledo DSC 823^e^ calorimeter equipped with a Julabo FT400 intracooler, was used in order to observe the curing reaction and the vitrification. For these TOPEM experiments, the temperature amplitude of the pulses was selected to be 1 K, with the switching time range from 15 to 30 s. All DSC curing experiments were performed with a dry nitrogen gas flow of 50 mL/min. The data evaluation was performed with the STARe software. The DSC was calibrated for both heat flow and temperature using indium. A small sample of about 8–10 mg was weighed into an aluminium pan, sealed, and immediately inserted into the DSC furnace, whereupon the curing experiment was immediately started. Non-isothermal scans were made at rates of 0.5 °C/min, 2 °C/min, and 10 °C/min over a temperature range from −65 °C to 200 °C, to determine the glass transition temperature, *T*_g0_, of the uncured sample and the heat of reaction, and were followed by a second scan at 10 °C/min to determine the glass transition temperature, *T*_g∞_, of the fully cured sample. The determination of the glass transition temperatures of the pure components was made by means of DSC experiments at 2 and 10 °C/min over the temperature range from −70 °C to 35 °C.

A dielectric analyser, DEA 2970 from TA Instruments, was used to measure the dielectric signals as a function of temperature and frequency. Dielectric measurements were performed using a ceramic single-surface cell of dimensions 20 × 25 mm based on a coplanar inter-digitated comb-like electrode design. The values of dielectric permittivity, ε′, and dielectric loss factor, ε″, were calculated from the resulting current and the induced phase angle shift, and the loss tangent, tan δ = ε″/ε′. The interval of data sampling was 1 s per point. The sample was spread on the electrode surface, covering the entire inter-digitated area. The non-isothermal curing measurements were performed at temperatures from −130 °C to 150 °C in a nitrogen atmosphere with a gas flow of 500 mL/min. The heating rates were 0.5 °C/min and 2 °C/min. The dielectric permittivity and the dielectric loss factor were measured at frequencies in the range from 0.1 Hz to 100 kHz. Subsequently, a second non-isothermal scan was made at a heating rate of 5 °C/min from 30 °C to 200 °C. Dielectric measurements for the cured ELP plaque, which measured 25 mm× 25 mm× 1.3 mm, were performed using a parallel plate sensor; the non-isothermal measurements were performed at temperatures from −130 °C to 180 °C and the heating rate was 2 °C/min.

Dynamic mechanical analysis (DMA) was carried out with the dynamic mechanic analyser DMA 861^e^ (Mettler-Toledo) in shear mode. The experiments for the pure components and for the uncured blends, ELP (viscous liquid samples), were performed using a special shear clamp, with a temperature range from −130 °C to 25 °C and a heating rate of 2 °C/min. A frequency range from 0.1 Hz to 100 Hz was used. The experiments for the cured ELP800 and ELP25000 samples, in the form of discs, were performed over the temperature range from −110 °C to 180 °C with a heating rate of 2 °C/min. The diameter and thickness of the ELP discs were 5.75 mm× 1.3 mm.

## 4. Conclusions

Hyperbranched poly(ethyleneimine) (HBPEI) samples of different molecular weights (MW), denoted PEI800 and PEI25000, and uncured blends of poly(ethyleneimine) with epoxy resin (DGEBA), denoted ELP800 and ELP25000, have been studied using DSC, DRS, and DMA techniques, and have been compared with earlier results obtained with an HBPEI of MW 2000. The average glass transition temperatures, *T*_g_, for the PEI800 and PEI25000 samples are −60 °C and −52 °C, respectively, increasing with the MW of the HBPEI, in agreement with the earlier result of −55 °C for PEI2000. The glass transition temperatures of the mixtures of HBPEI with the epoxy resin in the stage prior to curing, *T*_g0_, exhibit values between −17 and −22 °C, which are very close to those of the epoxy resin and only slightly influenced by the MW of the HBPEI. Using the TGA technique, it has been shown that the hyperbranched PEI2000 is thermally more stable than PEI800 and PEI25000, while the thermal stabilities of the mixtures of HBPEI and epoxy resin do not vary significantly with MW, either prior to curing or in the fully cured state.

By DSC, it was observed that the MW of the HBP in the ELP mixtures influences the value of the heat of reaction (Δ*H* for ELP800 > Δ*H* for ELP2000 > Δ*H* for ELP25000) and the peak exotherm temperature, with the curing reaction in ELP800 being advanced with respect to ELP2000 and ELP25000. These results indicate that ELP800 is more reactive than ELP25000 and ELP2000, as a consequence of PEI800 containing a greater number of H equivalents per gram of HBPEI.

Dielectric relaxation spectroscopy (DRS) shows three dipolar relaxations in all PEI samples. The γ-relaxation is attributed to the mobility of the end groups (NH_2_) present on the branches. The average value of the activation energy for this relaxation is 44 ± 2 kJ/mol for all HBPEIs, independent of the MW. The β-relaxation is attributed to the mobility of the branches. The MW of the HBPEI influences the temperature range in which the β-relaxation appears, being displaced to higher temperatures the higher the MW is. The average activation energy for the PEI800 and PEI25000 systems was 169 ± 20 kJ/mol, which is much higher than other values reported in the literature for other HBPEIs; the value for PEI2000 could not be reliably determined as it appeared only as a shoulder on the α-relaxation. The frequency-dependent dipolar α-relaxation, occurring at a higher temperature, is associated with the glass transition of the material, and is dependent on the MW of the HBPEI. A shoulder on the high temperature side of the α-relaxation suggests the existence of an ionic relaxation, which becomes more pronounced and extends over a wider frequency range with increasing MW of the PEI.

In the uncured ELP mixtures, γ-, β-, and α-relaxations are all detected by DRS, while only β- and α-relaxations are observed by DMA. The **γ**-relaxations of ELP800, ELP2000, and ELP25000 are independent of the MW of the HBPEI. The β-relaxation in these mixtures nearly always appears as a shoulder on the α-relaxation; only for the ELP800 mixture, and only then for the lowest frequencies, is it possible to analyse the frequency dependence of the peak temperature. The α-relaxation shows a significantly different behaviour according to the MW of the sample: for ELP800 and ELP2000, it splits into two peaks, particularly at low frequencies, which correspond to relaxations in the two phases identified by optical microscopy, while in ELP25000, only a single relaxation is observed. At temperatures above the α-relaxation, a peak is observed in both ELP800 and ELP25000, and ELP2000, being more pronounced in ELP800 and ELP2000 than in ELP25000, and corresponds to the curing reaction of the ELP system. The peak temperature occurs between 0 °C and 60 °C and is ionic in origin. In the curing step, ELP800, ELP2000, and ELP25000 all show a dipolar relaxation peak, corresponding to vitrification, in the range between 50 °C and 70 °C, as detected by TOPEM. This relaxation is more pronounced the higher the frequency is and the lower the MW of the HBPEI is. In the post-curing step, the fully cured ELP800, ELP2000, and ELP25000 samples show both β- and α-relaxations by DRS and DMA. The secondary β-relaxation is fairly well defined throughout the frequency range and over a wide temperature range. The temperature at which this relaxation appears, for each frequency, is essentially independent of the MW of the HBPEI. This relaxation is related to the mobility of segments of the branches between crosslinks. The α-relaxation by DRS is observed only as a shoulder at about 70 °C as a consequence of an overlap with conductivity effects, whereas by DMA it is a clear peak, and the apparent activation energy is independent of the MW of the HBPEI.

## Figures and Tables

**Table 1:** Results of the analysis of thermal degradation of the samples studied. The degradation temperatures are evaluated as: temperature of 5% weight loss, *T*_D5%_; the onset temperature, *T*_onset_; the peak temperature of the DTGA curve.

Sample	Heating Rate (°C/min)	*T*_D5%_ (°C)	*T*_onset_ (°C)	DTGA Peak (°C)
PEI800	2	200	322	362
	10	249	365	389
PEI2000 [69]	2	272	352	369
	10	301	379	399
PEI25000	2	283	311	336
	10	309	342	367
ELP800	2	305	317	343
	10	336	339	365
ELP2000 [69]	2	303	317	342
	10	305	347	374
ELP25000	2	310	314	333
	10	329	346	374

**Table 2:** DSC results for the HBPs, PEI800, PEI25000, and PEI2000, and for the ELP800, ELP25000, and ELP2000 systems (HBPEI + epoxy resin) at different heating rates.

Sample	Heating Rate (°C/min)	DSC (Conventional)					DSC (TOPEM)					DSC Cured films 50 °C, 3 h +140 °C, 3 h
		*T*_g0_ (°C)	Δ*H* (J/g)	Δ*H* (kJ/ee)	*T*_p_ (°C)	*T*_g∞,10_ (°C) ^a^	Δ*H* (J/g)	Δ*H* (kJ/ee)	*T*_p_ (°C)	*T*_v_ (°C)	*T*_g∞,2_ (°C) ^b^	*T*_g∞,10_ (°C) ^c^
PEI800	2	−62	-	-	-	-	-	-	-	-	-	-
	10	−58	-	-	-	-	-	-	-	-	-	-
PEI2000 [69]	2	−55	-	-	-	-	-	-	-	-	-	-
	10	−54	-	-	-	-	-	-	-	-	-	-
PEI25000	2	−53	-	-	-	-	-	-	-	-	-	-
	10	−52	-	-	-	-	-	-	-	-	-	-
Epoxy [69]	2	−17	-	-	-	-	-	-	-	-	-	-
	10	−16	-	-	-	-	-	-	-	-	-	-
ELP800	0.5	−22	453	99.9	55	113	390	86.2	54	67	113	-
	2	−18	432	95.0	72	98	-	-	-	-	-	105
	10	−20	434	95.9	101	101	-	-	-	-	-	112
ELP2000 [69]	0.5	−18	389	87.2	57	121	331	74.2	54	65	115	-
	2	−18	409	91.7	76	125	-	-	-	-	-	112
ELP25000	0.5	−20	380	86.4	59	116	361	82.0	57	68	118	-
	2	−20	386	87.6	79	114	-	-	-	-	-	112
	10	−18	354	80.6	103	105	-	-	-	-	-	118

^a^ Second scan at heating rate of 10 °C/min; ^b^ Second scan at heating rate of 2 °C/min; ^c^ First scan at heating rate of 10 °C/min.

**Table 3:** Results of the analysis of dielectric measurements, DRS, for the different samples studied: HBPEIs, epoxy, and uncured ELP systems.

Sample	Heating Rate (°C/min)	Relaxations, DRS Results		
		γ	β	α
		*E*_a_ (kJ/mol)	*E*_a_ (kJ/mol)	*E*_a app_ (kJ/mol)
PEI800	0.5	46.2	181	71.3
	2.0	45.8	155	66.1
PEI2000 [69]	0.5	42.0	-	78.1
	2.0	41.0	-	63.3
PEI25000	0.5	45.6	171	58.8
	2.0	41.7	169	59.4
EPOXY [69]	2.0	27.7	79.9	306
ELP800	0.5	29.6	73.5	272
	2.0	28.5	70.3	280
ELP2000 [69]	0.5	36.0	-	302
	2.0	27.3	-	263
ELP25000	0.5	35.6	-	310
	2.0	31.4	-	334

**Table 4:** Activation energy results obtained from dielectric spectroscopy (DRS) for fully cured ELP800, ELP25000, and ELP2000 samples.

Sample	Heating Rate (°C/min)	DRS Relaxations	
		β	α
		*E*_a_ (kJ/mol)	*E*_a app_ (kJ/mol)
ELP800	2	53.1	128
ELP2000	2	53.8	158
ELP25000	2	57.6	159

**Table 5:** Activation energy results obtained from dynamic mechanical (DMA) measurements for uncured and fully cured samples.

Sample	Heating Rate (°C/min)	Relaxations, DMA Uncured Samples		Relaxations, DMA Fully Cured Samples	
		β	α	β	α
		*E*_a_ (kJ/mol)	*E*_a app_ (kJ/mol)	*E*_a_ (kJ/mol)	*E*_a app_ (kJ/mol)
PEI800	2.0	194	224	-	-
PEI2000 [69]	2.0	-	209	-	-
PEI25000	2.0	192	234	-	-
EPOXY	2.0	89.0	358	-	-
ELP800	2.0	69.1	364	62.1	319
ELP2000 [69]	2.0	79.0	344	57.8	323
ELP25000	2.0	84.2	367	64.5	332

**Table 6:** Characteristics of the HBPEI samples.

Sample	Amine Ratio ^a^	Equivalent H Mass (g/equiv H) ^b^	Equivalent N Mass (g/equiv N) ^b^	Degree of Branching ^c^ (DB)
PEI800 ^d^	1:0.82:0.7	33.7	40.4	0.56
PEI25000 ^d^	1:1.2:0.76	38.8	42.0	0.56

^a^ Ratio of primary:secondary:tertiary amine groups. ^b^ The H equivalent mass (g/equiv H) is defined with respect to the number of reactive sites for epoxy-amine addition and the N equivalent mass (g/equiv N) is defined with respect to the total number of amine groups. ^c^ Calculated using the secondary amine (linear units) and tertiary amine (branching units) ratio according to the expression in ref. [87]. ^d^ The amine ratio and molecular mass data have been obtained from the supplier. The PEI800 and PEI25000 data (H equivalent mass and N equivalent mass) have been supplied by X. Fernandez-Francos [66].

## References

[1] Gao C., Yan D. (2004). Hyperbranched polymers: From synthesis to applications. Prog. Polym. Sci..

[2] Voit B. (2000). New developments in hyperbranched polymers. J. Polym. Sci. Part A Polym. Chem..

[3] Malmström E., Hult A. (1997). Hyperbranched polymers: A review. J. Macromol. Sci. Rev. Macromol. Chem. Phys..

[4] Yates C.R., Hayes W. (2004). Synthesis and applications of hyperbranched polymers. Eur. Polym. J..

[5] Hawker C.J., Lee R., Fréchet J.M.J. (1991). One-step synthesis of hyperbranched dendritic polyesters. J. Am. Chem. Soc..

[6] Holter D., Burgath A., Frey H. (1997). Degree of branching in hyperbranched polymers. Acta Polym..

[7] Jikei M., Kakimoto M. (2001). Hyperbranched polymers: A promising new class of materials. Prog. Polym. Sci..

[8] Scholl M., Kadlecova Z., Klok H.A. (2009). Dendritic and hyperbranched polyamides. Prog. Polym. Sci..

[9] Massa D.J., Shriner K.A., Turner S.R., Voit B. (1995). Novel blends of hyperbranched polyesters and linear polymers. Macromolecules.

[10] Mecking S., Thomann R., Frey H., Sunder A. (2000). Preparation of catalytically active palladium nanoclusters in compartments of amphiphilic hyperbranched polyglycerol. Macromolecules.

[11] Stiriba S.E., Kautz H., Frey H. (2002). Hyperbranched molecular nanocapsules: Comparison of the hyperbranched architecture with the perfect linear analogue. J. Am. Chem. Soc..

[12] Slagt M.Q., Stiriba S.E., Gebbink R.J.M.K., Kautz H., Frey H., van Koten G. (2002). Encapsulation of hydrophilic pincer-platinum (II) complexes in amphiphilic hyperbranched polyglycerol nanocapsules. Macromolecules.

[13] Hedrick J.L., Hawker C.J., Miller R.D., Twieg R., Srinivasan S.A., Trollsas M. (1997). Structure control in organic–inorganic hybrids using hyperbranched high-temperature polymers. Macromolecules.

[14] Sun Q.H., Xu K.T., Lam J.W.Y., Cha J.A.K., Zhang X.X., Tang B.Z. (2001). Nanostructured magnetoceramics from hyperbranched polymer precursors. Mater. Sci. Eng. C.

[15] Cosulich M.E., Russo S., Pasquale S., Mariani A. (2000). Performance evaluation of hyperbranched aramids as potential supports for protein immobilization. Polymer.

[16] Lim Y.B., Kim S.M., Lee Y., Lee W.K., Yang T.G., Lee M.J., Suh H., Park J.S. (2001). Cationic hyperbranched poly(amino ester): A novel class of DNA condensing molecule with cationic surface, biodegradable three-dimensional structure, and tertiary amine groups in the interior. J. Am. Chem. Soc..

[17] Frey H., Haag R. (2002). Dendritic polyglycerol: A new versatile biocompatible material. J. Biotechnol..

[18] Kim Y.H., Webster O.W. (1992). Hyperbranched polyphenylenes. Macromolecules.

[19] Hong Y., Cooper-White J.J., Mackay M.E., Hawker C.J., Malmström E., Rehnberg N. (1999). A novel processing aid for polymer extrusion: Rheology and processing of polyethylene and hyperbranched polymer blends. J. Rheol..

[20] Hong Y., Coombs S.J., Cooper-White J.J., Mackay M.E., Hawker C.J., Malmström E., Rehnberg N. (2000). Film blowing of linear low-density polyethylene blended with a novel hyperbranched polymer processing aid. Polymer.

[21] Mulkern T.J., Tan N.C.B. (2000). Processing and characterization of reactive polystyrene/hyperbranched polyester blends. Polymer.

[22] Jang J., Oh J.H., Moon S.I. (2000). Crystallization behavior of poly(ethylene terephthalate) blended with hyperbranched polymers: The effect of terminal groups and composition of hyperbranched polymers. Macromolecules.

[23] Ratna D., Simon G.P. (2001). Thermomechanical properties and morphology of blends of a hydroxy-functionalized hyperbranched polymer and epoxy resin. Polymer.

[24] Tang L.M., Qu T., Tuo X.L., Zhang X.L., Liu D.S. (2002). Synthesis, morphology and application of alkylaryl hyperbranched polyesters. Polym. J..

[25] Johansson M., Hult A. (1995). Synthesis, characterization and UV curing of acrylate functional hyperbranched polyester resins. J. Coat. Technol..

[26] Emrick T., Chang H.T., Frechet J.M.J., Woods J., Baccei L. (2000). Hyperbranched aromatic epoxies in the design of adhesive materials. Polym. Bull..

[27] Liu H., Wilen C.E., Skrifvars M. (2000). Reaction of epoxy resin and hyperbranched polyacids. J. Polym. Sci. Part A Polym. Chem..

[28] Schmaljohann D., Pötschke P., Hässler R., Voit B.I., Froehling P.E., Mostert B., Loontjens J.A. (1999). Blends of amphiphilic hyperbranched polyesters and different polyolefins. Macromolecules.

[29] Burkinshaw S.M., Froehling P.E., Mignanelli M. (2002). The effect of hyperbranched polymers on the dyeing of polypropylene fibres. Dyes Pigments.

[30] Xu J., Wu H., Mills O.P., Heiden P.A. (1999). A morphological investigation of thermosets toughened with novel thermoplastics. I. Bismaleimide modified with hyperbranched polyester. J. Appl. Polym. Sci..

[31] Gopala A., Wu H., Xu J., Heiden P. (1999). Investigation of readily processable thermoplastic-toughened thermosets. IV. BMIs toughened with hyperbranched polyester. J. Appl. Polym. Sci..

[32] Wu H., Xu J., Liu Y., Heiden P. (1999). Investigation of readily processable thermoplastic toughened thermosets. V. Epoxy resin toughened with hyperbranched polyester. J. Appl. Polym. Sci..

[33] Boogh L., Pettersson B., Manson J.A.E. (1999). Dendritic hyperbranched polymers as tougheners for epoxy resins. Polymer.

[34] Mezzenga R., Boogh L., Pettersson B., Manson J.A.E. (2000). Chemically induced phase separated morphologies in epoxy resin hyperbranched polymer blends. Macromol. Symp..

[35] Mezzenga R., Boogh L., Manson J.A.E. (2001). A review of dendritic hyperbranched polymer as modifiers in epoxy composites. Compos. Sci. Technol..

[36] Mezzenga R., Plummer C.J.G., Boogh L., Manson J.A.E. (2001). Morphology build-up in dendritic hyperbranched polymer modified epoxy resins: Modelling and characterization. Polymer.

[37] Gryshchuk O., Jost N., Karger-Kocsis J. (2002). Toughening of vinylester–urethane hybrid resins by functional liquid nitrile rubbers and hyperbranched polymers. Polymer.

[38] Gryshchuk O., Jost N., Karger-Kocsis J. (2002). Toughening of vinylester-urethane hybrid resins through functionalized polymers. J. Appl. Polym. Sci..

[39] Jannerfeldt G., Boogh L., Manson J.A.E. (2001). The morphology of hyperbranched polymer compatibilized polypropylene/polyamide 6 blends. Polym. Eng. Sci..

[40] Star A., Stoddart J.F. (2002). Dispersion and solubilization of single-walled carbon nanotubes with a hyperbranched polymer. Macromolecules.

[41] Haag R. (2001). Dendrimers and hyperbranched polymers as high-loading supports for organic synthesis. Chem. Eur. J..

[42] Klein Gebbink R.J.M., Kruithof C.A., Van Klink G.P.M., Van Koten G. (2002). Dendritic supports in organic synthesis. J. Biotechnol..

[43] Johansson M., Malmström E., Jansson A., Hult A. (2000). Novel concept for low temperature curing powder coatings based on hyperbranched polyesters. J. Coat. Technol..

[44] Van Benthem R.A.T.M. (2000). Novel hyperbranched resins for coating applications. Prog. Org. Coat..

[45] Manczyk K., Szewczyk P. (2002). Highly branched high solids alkyd resins. Prog. Org. Coat..

[46] Zhu S.W., Shi W.F. (2002). Flame retardant mechanism of hyperbranched polyurethane acrylates used for UV curable flame retardant coatings. Polym. Degrad. Stab..

[47] Zhu S.W., Shi W.F. (2002). Synthesis and photopolymerization of hyperbranched polyurethane acrylates applied to UV curable flame retardant coatings. Polym. Int..

[48] Lange J., Stenroos E., Johansson M., Malmström E. (2001). Barrier coatings for flexible packaging based on hyperbranched resins. Polymer.

[49] Lackowski W.M., Ghosh P., Crooks R.M. (1999). Micron-scale patterning of hyperbranched polymer films by micro-contact printing. J. Am. Chem. Soc..

[50] Ghosh P., Crooks R.M. (1999). Covalent grafting of a patterned, hyperbranched polymer onto a plastic substrate using microcontact printing. J. Am. Chem. Soc..

[51] Ghosh P., Amirpour M.L., Lackowski W.M., Pishko M.V., Crooks R.M. (1999). A simple lithographylic approach for preparing patterned, micron-scale corrals for controlling cell growth. Angew. Chem. Int. Ed..

[52] Aoki A., Ghosh P., Crooks R.M. (1999). Micrometer-scale patterning of multiple dyes on hyperbranched polymer thin films using photoacid-based lithography. Langmuir.

[53] Ghosh P., Lackowski W.M., Crooks R.M. (2001). Two new approaches for patterning polymer films using templates prepared by microcontact printing. Macromolecules.

[54] Crooks R.M. (2001). Patterning of hyperbranched polymer films. Chem. Phys. Chem..

[55] Ratna D., Simon G.P. (2001). Thermal and mechanical properties of a hydroxyl-functional dendritic hyperbranched polymer and trifunctional epoxy resin blends. Polym. Eng. Sci..

[56] Oh J.H., Jang J.S., Lee S.H. (2001). Curing behavior of tetrafunctional epoxy resin/hyperbranched polymer system. Polymer.

[57] Foix D., Yu Y.F., Serra A., Ramis X., Salla J.M. (2009). Study on the chemical modification of epoxy/anhydride thermosets using a hydroxyl terminated hyperbranched polymer. Eur. Polym. J..

[58] Fernandez-Francos X., Salla J.M., Cadenato A., Morancho J.M., Serra A., Mantecón A., Ramis X. (2009). A new strategy for controlling shrinkage of DGEBA resins cured by cationic copolymerization with hydroxyl-terminated hyperbranched polymers and ytterbium triflate as an initiator. J. Appl. Polym. Sci..

[59] Sangermano M., Priola A., Malucelli G., Bongiovanni R., Quaglia A., Voit B., Ziemer A. (2004). Phenolic hyperbranched polymers as additives in cationic photopolymerization of epoxy systems. Macromol. Mater. Eng..

[60] Sangermano M., Malucelli G., Bongiovanni R., Priola A., Harden A. (2005). Investigation on the effect of the presence of hyperbranched polymers on thermal and mechanical properties of an epoxy UV-cured system. Polym. Int..

[61] Johansonn M., Glauser T., Rospo G., Hult A. (2000). Radiation curing of hyperbranched polyester resins. J. Appl. Polym. Sci..

[62] Ratna D., Becker O., Krishnamurthy R., Simon G.P., Varley R.J. (2003). Nanocomposites based on a combination of epoxy resin, hyperbranched epoxy and a layered silicate. Polymer.

[63] Cortés P., Fraga I., Calventus Y., Román F., Hutchinson J.M., Ferrando F. (2014). A new epoxy-based layered silicate nanocomposite using a hyperbranched polymer: Study of the curing reaction and nanostructure development. Materials.

[64] Shiravand F., Fraga I., Cortés P., Calventus Y., Hutchinson J.M. (2014). Thermal analysis of polymer layered silicate nanocomposites: Identification of nanostructure development by DSC. J. Therm. Anal. Calorim..

[65] Santiago D., Fernandez-Francos X., Ramis X., Salla J.M., Sangermano M. (2011). Comparative curing kinetics and thermal-mechanical properties of DGEBA thermosets cured with a hyperbranched poly(ethyleneimine) and an aliphatic triamine. Thermochim. Acta.

[66] Fernandez-Francos X., Santiago D., Ferrando F., Ramis X., Salla J.M., Serra A., Sangermano M. (2012). Network structure and thermomechanical properties of hybrid DGEBA networks cured with 1-methylimidazole and hyperbranched poly(ethyleneimine)s. J. Polym. Sci. Part B Polym. Phys..

[67] Corezzi S., Beiner M., Huth H., Schröter K., Capaccioli S., Casalini R., Fioretto D., Donth E. (2002). Two crossover regions in the dynamics of glass forming epoxy resins. J. Chem. Phys..

[68] Beiner M., Ngai K.L. (2005). Interrelation between primary and secondary relaxations in polymerizing systems based on epoxy resins. Macromolecules.

[69] Roman F., Colomer P., Calventus Y., Hutchinson J.M. (2016). Molecular mobility in hyperbranched polymers and their interaction with an epoxy matrix. Materials.

[70] Mangion M.B.M., Johari G.P. (1990). Relaxations of thermosets. 3. Sub-Tg dielectric relaxations of bisphenol-A based epoxide cured with different cross-linking agents. J. Polym. Sci. Part B Polym. Phys..

[71] Shimbo M., Ochi M., Iesako H. (1984). Mechanical relaxation mechanism of epoxide resins cured with acid anhydrides. J. Polym. Sci. Part B Polym. Phys..

[72] Ochi M., Yoshizumi M., Shimbo M. (1987). Mechanical and dielectric relaxations of epoxide resins containing the spiro-ring structure. 2. Effect of the introduction of methoxy branches on low temperature relaxations of epoxide resins. J. Polym. Sci. Part B Polym. Phys..

[73] Maroulas P., Kripotou S., Sysel P., Hobzova R., Kotek J., Pissis P. (2006). Molecular dynamics in hyperbranched polyimides cross-linked with ethylene glycol diglycidyl ether. J. Non-Cryst. Sol..

[74] Zhu P.W., Zheng S., Simon G. (2001). Dielectric relaxations in a hyperbranched polyester with terminal hydroxyl groups: Effects of generation number. Macromol. Chem. Phys..

[75] Fraga I., Montserrat S., Hutchinson J.M. (2007). TOPEM, a new temperature modulated DSC technique. Application to the glass transition of polymers. J. Therm. Anal. Calorim..

[76] Van Assche G., Van Hemelrijck A., Rahier H., Van Mele B. (1996). Modulated differential scanning calorimetry: Non-isothermal cure, vitrification, and devitrification of thermosetting systems. Thermochim. Acta.

[77] Fraga I., Montserrat S., Hutchinson J.M. (2010). Vitrification and devitrification during the non-isothermal cure of a thermoset. Theoretical model and comparison with calorimetric experiments. Macromol. Chem. Phys..

[78] Fraga I., Hutchinson J.M., Montserrat S. (2010). Vitrification and devitrification during the non-isothermal cure of a thermoset. J. Therm. Anal. Calorim..

[79] Malmström E., Liu F., Boyd R.H., Hult A., Gedde U.W. (1994). Relaxation processes in hyperbranched polyesters. Polym. Bull..

[80] Malmström E., Hult A., Gedde U.W., Liu F., Boyd R.H. (1997). Relaxation processes in hyperbranched polyesters: Influence of terminal groups. Polymer.

[81] Roman F., Colomer P., Calventus Y., Hutchinson J.M. (2017). Study of the molecular dynamics of multiarm star polymers with a poly(ethyleneimine) core and poly(lactide) multiarms. Materials.

[82] Ngai K.L., Paluch M. (2004). Classification of secondary relaxation in glass-formers based on dynamic properties. J. Chem. Phys..

[83] Garcia-Bernabé A., Diaz-Calleja R., Haag R. (2006). Broadband dielectric spectroscopy studies of hyperbranched polyglycerols. Macromol. Chem. Phys..

[84] Turky G., Sangoro J.R., Rehim M.A., Kremer F. (2010). Secondary relaxations and electrical conductivity in hyperbranched polyester amides. J. Polym. Sci. Part B Polym. Phys..

[85] Fraga I., Montserrat S., Hutchinson J.M. (2008). Vitrification during the isothermal cure of thermosets: Comparison of theoretical simulations with temperature-modulated DSC and dielectric analysis. Macromol. Chem. Phys..

[86] Tombari E., Salvetti G., Johari G.P. (2000). The temperature and polymerization effects on the relaxation time and conductivity, and the evolution of the localized motions. J. Chem. Phys..

[87] Sunder A., Hanselmann R., Frey H., Mulhaupt R. (1999). Controlled synthesis of hyperbranched polyglycerols by ring-opening multibranching polymerization. Macromolecules.

